# Ferroptosis: whERe is the critical site of lipid peroxidation?

**DOI:** 10.3389/fcell.2023.1179245

**Published:** 2023-05-18

**Authors:** Wan Seok Yang

**Affiliations:** Department of Biological Sciences, St. John’s University, New York, NY, United States

**Keywords:** ferroptosis, endoplasmic reticulum, Raman imaging, lipid perodixation, FINO2, D-PUFA, ike, RSL3

Ferroptosis is a regulated form of cell death that is driven by a lethal accumulation of lipid peroxides in cell membranes (Dixon et al., 2012). Recent studies have revealed the involvement of ferroptosis in various physiological and pathological conditions (Stockwell, 2022). Understanding the precise mechanism of ferroptosis is necessary to explain this phenomenon in various contexts.

Following the initial observation that lipid peroxides in cell membranes “execute” ferroptotic cell death, researchers have reported a more specific biochemical nature of these peroxides. For example, a genome-wide CRISPR screening identified acyl-CoA synthetase long-chain family member 4 (ACSL4) as an essential gene for executing ferroptosis, revealing that the ACSL4-mediated loading of the PUFAs to the membrane phospholipids is a critical event for executing ferroptosis (Doll et al., 2017). Conversely, unloaded, free PUFAs within cells were found to be neutral in modulating ferroptosis sensitivity. In addition, LC-MS/MS-based lipidomics analysis showed that phosphatidylethanolamines (PEs) containing polyunsaturated fatty acids (PUFAs) were more susceptible to oxidative damage during ferroptosis compared to other phospholipids in cell membranes (Kagan et al., 2017). Later, another genome-wide CRISPR screening discovered ether-phospholipid with PUFAs as a distinct functional lipid class mediating ferroptosis (Zou et al., 2020).

Despite significant progress in identifying and refining lipid classes relevant to ferroptosis, there remined the question of whether specific subcellular locations of lipids that are more susceptible to ferroptosis initiation or play more important roles in ferroptosis execution. A recent study by von Krusenstiern and others (von Krusenstiern et al., 2023) addressed this question using fluorescence and stimulated Raman scattering (SRS) imaging techniques (Figure 1). Compared to fluorescent tags, Raman active tags offer an advantage in monitoring the subcellular location of small molecules, such as drugs or metabolites, due to their relatively tiny size (Shen et al., 2019).

**FIGURE 1:**
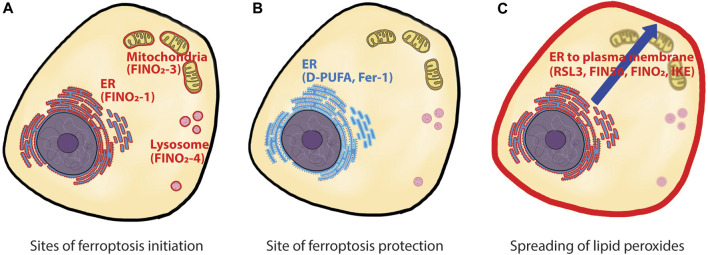
Site of action for ferroptosis initiation, protection, and spreading. **(A)** ferroptosis can be initiated from multiple sites within cells. Three ferroptosis inducers, FINO_2_-1, FINO_2_-3, and FINO_2_-4 initiated ferroptosis from different locations, namely, ER, mitochondria, and lysosome. **(B)** D-PUFA and Fer-1, the two ferroptosis inhibitors, reside in ER and suppress ferroptosis initiated from all locations. **(C)** time-lapse observation of ferroptosis showed initial lipid peroxidation in ER, then a late lipid peroxidation in the plasma membrane. How the lipid peroxides spread from ER to the plasma membrane is unknown. ER: endoplasmic reticulum, D-PUFA: deuterated polyunsaturated fatty acid, Fer-1: ferrostatin-1..

The authors first examined the cellular localization of deuterated-PUFA (D-PUFA), a specific ferroptosis inhibitor (von Krusenstiern et al., 2023; Yang et al., 2016). Since deuterium is a Raman active tag, there was no need to modify D-PUFA further to visualize it within cells. SRS imaging revealed that D-PUFAs were primarily located in perinuclear regions and abundant puncta structures in the cells, and subsequent fluorescence imaging with Nile Red confirmed the puncta structures as lipid droplets. Treatment of cells with diglyceride acyltransferase (DGAT) inhibitors removed these lipid droplets. However, the absence of lipid droplets did not affect D-PUFA’s anti-ferroptotic activity, suggesting that lipid droplets are not functionally relevant to ferroptosis. Further analysis using fluorescence microscopy with ER-tracker Green dye revealed that the perinuclear region was the endoplasmic reticulum (ER), indicating that the ER is another cellular location where D-PUFAs mainly reside. Unlike lipid droplets, however, there was no straightforward way to alter the surface area of ER using pharmacological or genetic reagents. For example, induction of ER-phagy (Mochida and Nakatogawa, 2022) was unsuccessful in the cell lines used in this study, despite extensive attempts. Instead, the authors treated cells with pro-ferroptotic PUFAs or anti-ferroptotic MUFAs and determined their cellular locations using SRS imaging. Both fatty acids were mainly located in the ER, with small percentages located in plasma membrane. The authors then overexpressed ACSL4 in the cells to increase the loading of pro-ferroptotic PUFAs or anti-ferroptotic MUFAs to the ER phospholipids. This approach demonstrated a solid correlation between the ER membrane composition and ferroptosis sensitivity, highlighting the ER as a site of ferroptosis modulation.

Next, the authors explored the site of action for FINO_2_, a canonical ferroptosis inducer, which directly oxidizes cellular iron and indirectly inhibits GPx4 to induce ferroptosis (Gaschler et al., 2018a). Structurally, FINO_2_ is a lipophilic compound that contains an endoperoxide moiety, allowing it to accumulate in cell membranes and induce lipid peroxidation directly at those locations. Taking advantage of this feature, the authors synthesized a series of FINO_2_ analogs with fluorescent tags that were distributed to different cellular locations to investigate whether ferroptosis can be initiated by oxidative damage in a particular cell membrane. The original FINO_2_ with a fluorescent tag displayed ER distribution and induced ferroptosis. Interestingly, FINO_2_ analogs distributed to mitochondria and lysosomes also induced ferroptosis, indicating that ferroptosis can be initiated from various organelles. More importantly, cells treated with mitochondrial and lysosomal FINO_2_ analogs were protected by ferrostatin-1 (Fer-1). A previous report demonstrated that Fer-1 primarily acted at the ER site, rather than in mitochondria and lysosomes which were dispensable to Fer-1’s anti-ferroptotic activity (Gaschler et al., 2018b). These data suggest that while ferroptosis can be initiated at multiple sites, the ER is the critical site for ferroptosis protection.

Lastly, the authors examined time-dependent changes in the location of lipid peroxidation using BODIPY™-C11 dye and fluorescence microscopy. Upon treatment with ferroptosis inducers, cells underwent initial lipid peroxidation events in the ER, followed by lipid peroxidation in the plasma membrane. This time-dependent change was observed in all four classes of ferroptosis inducers (RSL3, FIN56, FINO_2_, or IKE). These findings complement a previous study, which reported that both erastin2 and RSL3 caused intracellular lipid peroxidation before the onset of lipid peroxidation in the plasma membrane (Magtanong et al., 2022). Altogether, these data strongly point towards the ER membrane as the most critical site for inducing ferroptosis.

In summary, the findings outlined above emphasized the essential role of the ER in ferroptotic cell death. The conclusion from the current research raises several intriguing questions. First, what makes the ER so critical in executing ferroptosis? Does the ER membrane contain a higher level of ferroptosis-relevant lipids compared to other organelles? Are there any microenvironmental factors in the ER that can affect ferroptosis? A refined lipidomics analysis with subcellular fractionation samples may provide answers to these questions. Second, how is the ferroptosis signal initiated from other organelles transduced to the ER? For example, it is unclear how the lipid peroxides in mitochondria or lysosomes spread to the ER when FINO_2_ analogs are used. This question also applies to the later stage of ferroptosis. How are the lipid peroxides in the ER transduced or transported to the plasma membrane before the onset of cell death? Could it be the conventional vesicle-mediated transport or some other mechanism? Third, can we find more specific regions within the ER, such as rough ER, smooth ER, ER lumen, or microdomains in the ER membrane, that are responsible for the initiation of ferroptosis? Lastly, what are the ER-specific genes that regulate ferroptosis? Xiaodong Wang’s group recently discovered that the ER-resident oxidoreductases POR and CYB5R1 produced initial hydrogen peroxides, which subsequently induced lipid peroxidation and ferroptosis (Yan et al., 2021). Future research focused on the ER should broaden our knowledge about the natural triggers of ferroptosis and help identify better target proteins for ferroptosis targeted therapy.

## References

[B1] Dixon S. J. Lemberg K. M. Lamprecht M. R. Skouta R. Zaitsev E. M. Gleason C. E. (2012). Ferroptosis: An iron-dependent form of nonapoptotic cell death. Cell 149, 1060–1072. 10.1016/j.cell.2012.03.042 22632970 PMC3367386

[B2] Doll S. Proneth B. Tyurina Y. Y. Panzilius E. Kobayashi S. Ingold I. (2017). ACSL4 dictates ferroptosis sensitivity by shaping cellular lipid composition. Nat. Chem. Biol. 13, 91–98. 10.1038/nchembio.2239 27842070 PMC5610546

[B3] Gaschler M. M. Andia A. A. Liu H. Csuka J. M. Hurlocker B. Vaiana C. A. (2018). FINO(2) initiates ferroptosis through GPX4 inactivation and iron oxidation. Nat. Chem. Biol. 14, 507–515. 10.1038/s41589-018-0031-6 29610484 PMC5899674

[B4] Gaschler M. M. Hu F. Feng H. Linkermann A. Min W. Stockwell B. R. (2018). Determination of the subcellular localization and mechanism of action of ferrostatins in suppressing ferroptosis. ACS Chem. Biol. 13, 1013–1020. 10.1021/acschembio.8b00199 29512999 PMC5960802

[B5] Kagan V. E. Mao G. Qu F. Angeli J. P. F. Doll S. Croix C. S. (2017). Oxidized arachidonic and adrenic PEs navigate cells to ferroptosis. Nat. Chem. Biol. 13, 81–90. 10.1038/nchembio.2238 27842066 PMC5506843

[B6] Magtanong L. Mueller G. D. Williams K. J. Billmann M. Chan K. Armenta D. A. (2022). Context-dependent regulation of ferroptosis sensitivity. Cell Chem. Biol. 29, 1409–1418.e6. 10.1016/j.chembiol.2022.06.004 35809566 PMC9481678

[B7] Mochida K. Nakatogawa H. (2022). ER-Phagy: Selective autophagy of the endoplasmic reticulum. EMBO Rep. 23, e55192. 10.15252/embr.202255192 35758175 PMC9346472

[B8] Shen Y. Hu F. Min W. (2019). Raman imaging of small biomolecules. Annu. Rev. Biophys. 48, 347–369. 10.1146/annurev-biophys-052118-115500 30892920

[B9] Stockwell B. R. (2022). Ferroptosis turns 10: Emerging mechanisms, physiological functions, and therapeutic applications. Cell 185, 2401–2421. 10.1016/j.cell.2022.06.003 35803244 PMC9273022

[B10] von Krusenstiern A. N. Robson R. N. Qian N. Qiu B. Hu F. Reznik E. (2023). Identification of essential sites of lipid peroxidation in ferroptosis Nat. Chem. Biol. 10.1038/s41589-022-01249-3 PMC10238648 36747055

[B11] Yan B. Ai Y. Sun Q. Ma Y. Cao Y. Wang J. (2021). Membrane damage during ferroptosis is caused by oxidation of phospholipids catalyzed by the oxidoreductases POR and CYB5R1. Mol. Cell 81, 355–369.e10. 10.1016/j.molcel.2020.11.024 33321093

[B12] Yang W. S. Kim K. J. Gaschler M. M. Patel M. Shchepinov M. S. Stockwell B. R. (2016). Peroxidation of polyunsaturated fatty acids by lipoxygenases drives ferroptosis. Proc. Natl. Acad. Sci. U. S. A. 113, E4966–E4975. 10.1073/pnas.1603244113 27506793 PMC5003261

[B13] Zou Y. Henry W. S. Ricq E. L. Graham E. T. Phadnis V. V. Maretich P. (2020). Plasticity of ether lipids promotes ferroptosis susceptibility and evasion. Nature 585, 603–608. 10.1038/s41586-020-2732-8 32939090 PMC8051864

